# Salvianolic Acid B Slows the Progression of Breast Cancer Cell Growth via Enhancement of Apoptosis and Reduction of Oxidative Stress, Inflammation, and Angiogenesis

**DOI:** 10.3390/ijms20225653

**Published:** 2019-11-12

**Authors:** Mohamed A. Katary, Rafik Abdelsayed, Abdulmohsin Alhashim, Mohamed Abdelhasib, Ahmed A. Elmarakby

**Affiliations:** 1 Department of Oral Biology & Diagnostic Sciences, Augusta University, Augusta, GA 30912, USA; rabdelsa@augusta.edu (R.A.); aalhashim@augusta.edu (A.A.); 2 Department of Pharmacology & Toxicology, Damanhour University, Damanhour 22516, Egypt; mohamedalaa_pharmacy@hotmail.com; 3 Department of Pharmacology & Toxicology, Mansoura University, Mansoura 35516, Egypt

**Keywords:** salvianolic acid b, ESC, apoptosis, oxidative stress, inflammation

## Abstract

Breast cancer is the current leading cause of cancer death in females worldwide. Although current chemotherapeutic drugs effectively reduce the progression of breast cancer, most of these drugs have many unwanted side effects. Salvianolic acid B (Sal-B) is a bioactive compound isolated from the root of Danshen Radix with potent antioxidant and anti-inflammatory properties. Since free radicals play a key role in the initiation and progression of tumor cells growth and enhance their metastatic potential, the current study was designed to investigate the antitumor activity of Sal-B and compare it with the antitumor activity of the traditional anticancer drug, cisplatin. In vitro, Sal-B decreased the human breast cancer adenocarcinoma (MCF-7) cells proliferation in a concentration and time dependent manner. In vivo and similar to cisplatin treatment, Sal-B significantly reduced tumor volume and increased the median survival when compared to tumor positive control mice group injected with Ehrlich solid carcinoma cell line (ESC). Sal-B decreased plasma level of malondialdehyde as a marker of oxidative stress and increased plasma level of reduced glutathione (GSH) as a marker of antioxidant defense when compared to control ESC injected mice. Either Sal-B or cisplatin treatment decreased tumor tissue levels of tumor necrosis factor (TNF-α), matrix metalloproteinase-8 (MMP-8), and Cyclin D1 in ESC treated mice. Contrary to cisplatin treatment, Sal-B did not decrease tumor tissue Ki-67 protein in ESC injected mice. Immunohistochemical analysis revealed that Sal-B or cisplatin treatment increased the expression of the apoptotic markers caspase-3 and P53. Although Sal-B or cisplatin significantly reduced the expression of the angiogenic factor vascular endothelial growth factor (VEGF) in ESC injected mice, only Sal-B reduced expression level of COX-2 in ESC injected mice. Our data suggest that Sal-B exhibits antitumor features against breast cancer cells possibly via enhancing apoptosis and reducing oxidative stress, inflammation, and angiogenesis.

## 1. Introduction

Breast cancer is the most frequently diagnosed cancer and the leading cause of cancer death in females worldwide, about 23% of the total cancer cases and 14% of the cancer deaths [1]. In the Unites States, breast cancer is the most common cancer in women and is the second highest lethal form of cancer [2,3]. Although incidence of breast cancer has been gradually decreasing in the last two decades, women in the United States still have a one in eight chance to develop breast cancer [2]. One of the main obstacles in the treatment of breast cancer is poor response to chemotherapeutic agents and anti-hormonal treatment in 1/3 of breast cancer patients due to lack of expression of estrogen receptor, progesterone receptor, and human epidermal growth factor receptor-2 which are classified as triple negative patients [4]. Ehrlich tumor is malignant neoplasia derived from breast carcinoma in mice, with aggressive proliferative rate and rapid development of the tumor [5]. Since Ehrlich tumor clinically resembles human tumor, the solid and the ascetic forms of this tumor are frequently utilized to evaluate the antitumor activity of natural products [6].

Natural products are currently attracting attention as potential cancer therapeutics [7]. Salvianolic acid B (Sal-B), isolated from the root of the Chinese herb Danshen Radix, is the most abundant bioactive hydrophilic ingredient and is used as a quality control ingredient and active marker for Salviae Miltiorrhizae products by the China National Pharmacopeia Council [8]. Sal-B is effective in many cardiovascular and neurodegenerative diseases and previous studies have shown that Sal-B has anti-inflammatory and anti-oxidative effects [8]. The number of studies suggest that the effectiveness and safety of Sal-B relative to non-steroidal anti-inflammatory drugs (NSAIDs) in treatment of inflammatory condition as Sal-B does not produce similar adverse effects of NSAIDs upon prolong use (cardiovascular disease, GIT discomfort, and renal failure) [9]. For example, Sal-B has shown to reduce brain damage and improve motor function after cerebral ischemia in rats [10]. In addition to its anti-oxidant and anti-inflammatory properties, the protective effects of Sal-B could be attributed to anti-apoptotic property, inhibition of platelet aggregation, and improvement of coronary circulation as well as reduction of angiogenesis [9]. 

In addition to the diverse effects of Sal-B in improving vascular function and repairing the endothelial function, Sal-B had preventive effects against cancer growth [11]. For example, Sal-B enhanced anticancer activity of arsenic trioxide in human hepatoma (HepG2) cells and human cervical cancer (HeLa) cells [12]. Sal-B also showed a chemo-preventive effects for head and neck squamous cell cancer [9,13] as well as a growth inhibitory effect against acute promyelocytic leukemia cells [14]. Recent studies have demonstrated that Sal-B was effective against human glioblastoma U87 cells via P38 activation-mediated reactive oxygen species generation [15]. Moreover, Sal-B exhibited high efficacy against triple negative breast cancer cells which lack expression of estrogen receptor, progesterone receptor, and human epidermal growth factor receptor-2 as Sal-B reduced cell viability, inhibited tumor cell growth, and enhanced apoptosis [4]. 

Cisplatin is one of the most potent antineoplastic drugs for treatment of wide spectrum of human malignancies including head, neck, lung, and breast cancers [16]. Cisplatin provides its anticancer effect via crosslinking DNA and interfering with mitotic cell division. Consequently, the damaged DNA provokes DNA repair mechanisms, which sequentially activate apoptosis [17]. Cisplatin is used as a standard reference antineoplastic drug [18]. However, its clinical efficacy and use is limited by the drug resistance and side effects [15]. Thus, there is an urgent need of alternative new anti-cancer drugs with a better efficiency and safety margin. In light of the above mentioned, the current study was designed to investigate the antitumor activity of Sal-B against Ehrlich solid carcinoma and compare it with cisplatin in female Swiss albino mice in addition to determining the potential antitumor mechanisms of Sal-B against Ehrlich solid carcinoma.

## 2. Results

**Figure 1:**
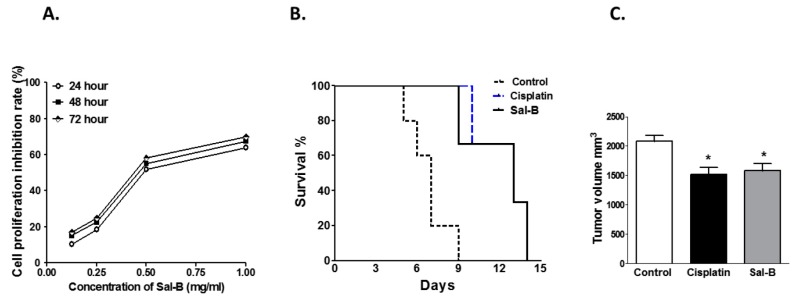
(**A**) Effect of salvianolic acid B (Sal-B) treatment with different concentrations (0.125, 0.25, 0.5, and 1 mg/mL, respectively) on Michigan Cancer Foundation-7 (MCF-7) breast cancer cells proliferation rate in vitro after 24, 48, and 72 h incubation. In vivo effect of Sal-B treatment (25 mg/kg, intraperitoneal (I.P.) daily injection for two weeks) or cisplatin (3.5 mg/kg, I.P. single injection) on survival percentage (**B**) and tumor volume (**C**) in Ehrlich solid carcinoma (ESC) injected mice (* *p* <0.05 versus control, *n* = 5–6/group).

**Figure 2:**
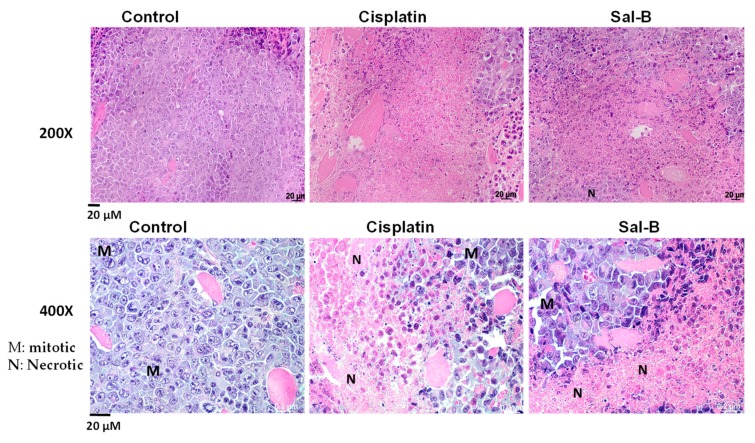
Representative images for H&E staining of tumor sections from ESC injected control, cisplatin, or Sal-B treated mice at 200× and 400× magnification power (N indicates necrotic area and M indicates mitotic area, *n* = 4/group).

The current study was designed to assess antitumor activity of Sal-B against breast cancer cells and compare it to the antitumor activity of cisplatin as a standard chemotherapeutic drug. As shown in Figure 1A, Sal-B significantly reduced the cell viability of MCF-7 cells in vitro in a concentration-and time-dependent manner. In Sal-B treated MCF-7 cells for 24, 48, and 72 h, the IC50 values of Sal-B were 4.5, 4.9, and 4.6 mg/mL, respectively. In vivo, ESC was injected S.C. in female mice to induce tumor similar to the clinical features of breast cancer. As shown by Kaplan–Meier’s survival analysis curve (Figure 1B), treatment with either Sal-B or cisplatin prolonged survival percentage of ESC injected mice when compared with ESC injected control group. Daily administration of Sal-B to mice bearing ESC resulted in a significant decrease in tumor volume (Figure 1C). The reduction of tumor volume in Sal-B treated mice group was similar to cisplatin treated mice (Figure 1C). Figure 2 showed that injection of ESC cells resulted in the development of Ehrlich solid tumor as manifested by sheets of neoplastic cells exhibiting marked nuclear atypia, multi-nucleation, and mitotic figures. Cisplatin treatment not only reduced the tumor size (Figure 1C) but also reduced the number of mitosis and increased necrosis (Figure 2). Similarly, Sal-B treatment was as effective as cisplatin in reducing the number of mitotic figures and increasing necrosis (Figure 2).

**Figure 3:**
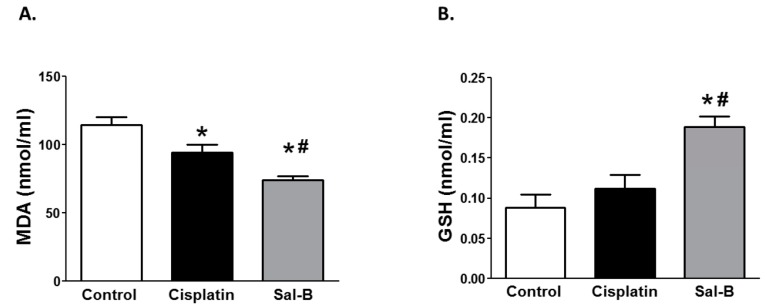
Effect of Sal-B (25 mg/kg, I.P. daily injection for two weeks) or cisplatin (3.5 mg/kg I.P.) treatment on the plasma levels of malondialdehyde (MDA) (**A**) and reduced glutathione (GSH) (**B**) as markers of oxidative stress and antioxidant defense system, respectively in ESC injected mice (* *p* < 0.05 (significant) when compared to control ESC injected mice, ^#^ *p* < 0.05 (significant) when compared to cisplatin treated ESC injected mice, *n* = 5–6/group).

Since oxidative stress plays a role in the pathogenesis and progression of tumor growth [15], we first assessed whether Sal-B could change oxidative stress in the plasma of ESC injected mice. As shown in Figure 3A, Sal-B or cisplatin treatment significantly decreased plasma malondialdehyde levels as a measure of oxidative stress in ESC injected mice (*p* < 0.05). However, only Sal-B treatment significantly increased plasma GSH levels, as a measure of antioxidant defense mechanism, in ESC injected mice (Figure 3B). 

**Figure 4:**
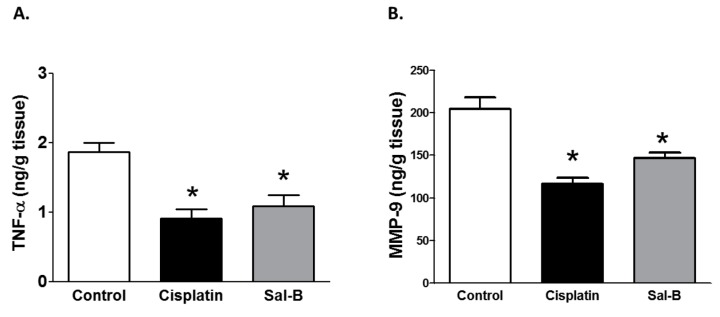
Effect of Sal-B (25 mg/kg, I.P. daily injection for two weeks) or cisplatin (3.5 mg/kg, I.P. on tumor tissue content of TNF-α (**A**) and MMP-9 (**B**) in ESC injected mice (* *p* <0.05 versus control ESC injected mice, *n* = 5–6/group).

**Figure 5:**
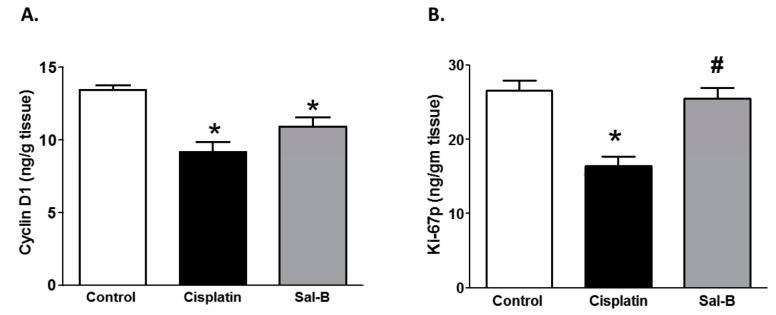
Effect of Sal-B (25 mg/kg, I.P. daily injection for two weeks) or cisplatin (3.5 mg/kg, I.P.) on tumor tissue content of Cyclin D1 (**A**) and Ki-67p (**B**) in ESC injected mice (* *p* <0.05 (significant) compared to control ESC injected mice and ^#^ *p* < 0.05 (significant) compared to cisplatin treated ESC injected mice; *n* = 5–6/group).

Inflammation also plays a role in the incidence and progression of tumor growth [19]. Sal-B or cisplatin treatment significantly reduced the tumor tissue level of the inflammatory cytokine TNF-α in ESC injected mice (Figure 4A). Since MMP-9 plays a crucial role in angiogenesis and tumor invasiveness, we further assessed the effect of Sal-B treatment on tumor tissue level of MMP-9. As shown in Figure 4B, either Sal-B or cisplatin also significantly decreased tumor tissue levels of MMP-9. Moreover, Sal-B or cisplatin treatment significantly decreased tumor tissue level of cyclin D1 in ESC injected mice which is required for the progression through the G1 phase of the cell cycle to induce cell migration and angiogenesis (Figure 5A). Only cisplatin treatment significantly decreases tumor tissue level of Ki-67 as a cellular marker of proliferation in ESC injected mice whereas Sal-B failed to provide a similar effect of cisplatin in ESC injected mice (Figure 5B).

**Figure 6:**
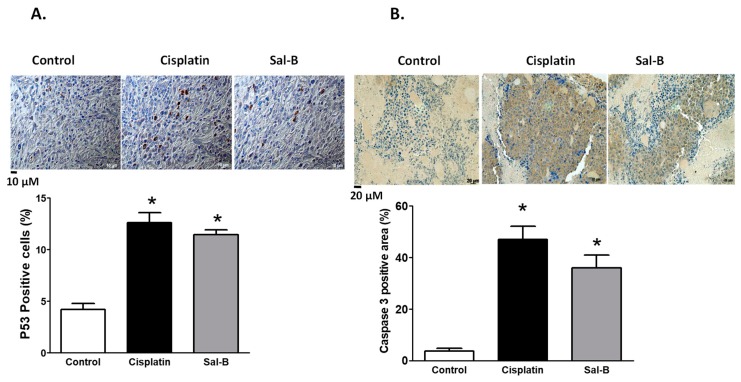
Representative images at 200× magnification and average percentage staining for P53 (**A**) as a pre-apoptotic marker and caspase-3 as an apoptotic marker (**B**) in ESC injected mice with or without cisplatin or Sal-B treatment (* *p* < 0.05 versus control ESC injected mice; *n* = 4/group).

P53 is a nuclear transcription factor with a pro-apoptotic function and is considered one of the classical type tumor suppressors [20]. Cisplatin or Sal-B treatment significantly increased expression level of p53 in tumor tissue of ESC injected mice (Figure 6A). Similarly, cisplatin or Sal-B treatment also significantly increased expression levels of caspase-3, the executioner of apoptosis [21], in tumor tissue of ESC injected mice (Figure 6B).

**Figure 7:**
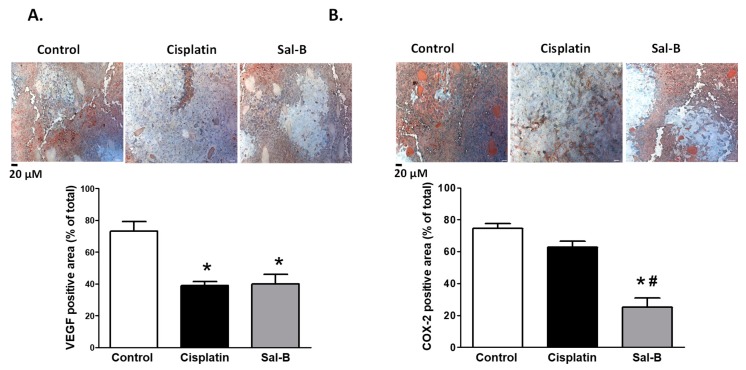
Representative images at 200× magnification and average percentage staining for the angiogenic markers VEGF (**A**) as and COX-2 (**B**) in ESC injected mice with or without cisplatin or Sal-B treatment (* *p* < 0.05 (significant) compared to control ESC injected mice and ^#^ *p* < 0.05 (significant) compared to cisplatin treated ESC injected mice; *n* = 4/group).

VEGF is known to be the key mediator for angiogenesis in cancer [22]. Our data suggest that cisplatin or Sal-B not only increased apoptosis but also decreased angiogenesis in ESC injected mice as VEGF expression levels were significantly reduced in tumor tissue of ESC injected mice treated with either cisplatin or Sal-B (Figure 7A). COX-2, the inducible form of cyclooxygenase (COX) is elevated in inflammatory and neoplastic diseases to produce prostaglandin metabolites (mainly PGE2), which is known to induce cell proliferation and promote tumor angiogenesis [23]. Previous studies have demonstrated that Sal-B decreased COX-2 expression and PGE2 production together with decreased growth of head and neck squamous cell carcinoma in vitro and in vivo [13]. Consistent with these data, Sal-B significantly decreased expression level of COX-2 in tumor tissue of ESC injected mice (Figure 7B). Although cisplatin treatment tended to decrease expression level of COX-2 in tumor tissue of ESC injected mice, this change was not significant (Figure 7B).

## 3. Discussion

The current study was designed to determine if Sal-B reduces the progression of cancer growth in ESC injected mice and compares its antitumor activity with cisplatin in addition to determine potential antitumor mechanisms of Sal-B. In vitro, the results of MTT assay showed that Sal-B significantly inhibited the proliferation of human breast cancer MCF-7 cells in concentration and time dependent manner. In parallel, in vivo Sal-B treatment prolonged mice survival and reduced tumor volume in ESC injected mice similar to cisplatin effect. In addition, histopathological examination demonstrated a reduction in number of mitosis and induction of necrosis in both cisplatin and Sal-B treated mice. The reduction of tumor volume in Sal-B treated mice as well as cisplatin treated mice was associated with reduced oxidative stress and inflammation. Although Sal-B or cisplatin treatment reduced tumor tissue level of cyclin D as marker of cell migration and angiogenesis, only cisplatin treatment reduced tumor tissue level of Ki-67 as a marker of cell proliferation in ESC injected mice. Sal-B or cisplatin treatment also increased expression of pro-apopotic marker P53 and apoptotic marker caspase-3 in tumor tissue of ESC injected mice. Increased tumor cells apoptosis with Sal-B or cisplatin treatment was associated with reduced tumor tissue expression level of the angiogenic marker VEGF. Sal-B was superior over cisplatin treatment in reducing tumor tissue expression level of the tumor angiogenic and inflammatory marker COX-2 in ESC injected mice. Our finding suggest that Sal-B exhibits antitumor effect against breast cancer cells via multiple mechanisms including anti-oxidant, anti-inflammatory, and anti-angiogenic properties besides enhancing tumor cells apoptosis. 

Previous studies suggest that Sal-B has anti-tumor activity against diverse number of cancer cell types such as mucosal cancer in golden hamster [24], human glioma [12], and acute promyelocytic leukemia cells [14]. Sal-B inhibited malignant transformation of oral precancerous lesion as it significantly decreased the incidence of oral squamous cell carcinoma in 7,12-dimethylbenz(a)anthracene (DMBA)-induced squamous cell carcinoma in hamster via inhibition of angiogenesis [25]. Consistent with these findings our in vitro data suggest that Sal-B reduced MCF-7 cells viability in a concentration and time dependent manner. 

Previous findings suggest that Sal-B treatment has protective effects against cardiovascular and neurodegenerative disorders via its antioxidant and free radical scavenging properties [26]. Reactive oxygen species (ROS) can disturb the equilibrium status of prooxidant/antioxidant reaction leading to an overall disruption of cellular function. Elevations of ROS are associated with many cardiovascular diseases [27]. For example, the protective effect of Sal-B on hydrogen peroxide-induced oxidative damage in SH-SY5Y cells were investigated by assessing cell viability assay, lactate dehydrogenase release, and levels of lipid peroxidation and Sal-B was superior over ginko biloba extract in its antioxidant efficiency [28]. Studies suggest that Sal-B had higher antioxidant capacity to neutralize free radical and higher scavenging activity than vitamin C [29]. Sal-B antioxidant reduced lipid peroxidation and improved antioxidant enzyme activity in ischemia-reperfusion injury [30]. Sal- B exhibited a protective effect against glutamate-induced excitotoxicity in pheochromocytoma PC12 cells via the inhibition of reactive oxygen species and caspase-3 pathway dependent apoptosis [31]. Sal-B also prevented arsenic trioxide induced cardiotoxicity via antioxidant and increase plasma levels of SOD and GSH [12]. Together, these studies suggest that the antioxidant effect of Sal-B could be the key mechanism in slowing progression of cardiovascular injury. 

Cancer cells contain high level of ROS due to oncogenic stimulation, increased metabolic activity, and/or mitochondrial dysfunction [32]. ROS are involved in a variety of different cellular processes ranging from apoptosis to carcinogenesis [33]. Activation of neutrophil and excessive formation of neutrophil extracellular traps (NETs) has been observed in the pathological condition of metastatic cancers. Sal-B has been demonstrated to interfere with NADPH oxidase-dependent NET formation thus reducing incidence of metastasis [34]. In hepatic stellate cells, Sal-B decreased ROS production and inhibited lipid peroxidation and cell proliferation via the inhibition of NADPH oxidase [35,36]. Consistent with previous findings, our data showed a significant reduction in plasma malondialdehyde and an increase in plasma GSH level in Sal-B treated group compared to tumor control group. Although cisplatin treatment also reduced plasma malondialdehyde levels in ESC injected mice, it did not increase plasma GSH as shown with Sal-B treatment. Consistent with our findings, Sal-B has shown previously to reduce H2O2-induced oxidative stress [37] and increased the antioxidant heme oxygenase-1 (HO-1) expression [38]. However, it is worthy to mention that fewer studies contradict with our findings and suggest that elevated ROS could be a biochemical basis for new pharmacological therapeutic strategy to kill cancer cells [15]. In fact and contrary to our results, these studies reported that Sal-B increased ROS generation and induced apoptotic cell death in U87 cells [15]. Sal-B also reversed tumor multidrug resistance in HCT-8/VCR cells, a multidrug resistant colorectal cancer cell line via increased reactive oxygen species which promote tumor cell apoptosis [39]. Thus, it seems that the effect of Sal-B on ROS production could be different based on cancer cell type. Overall, these data suggest that modulating levels of ROS contributes to anticancer effect of Sal-B.

Chronic inflammation increases the risk of many types of cancer and inflammatory cytokines are present in tumors microenvironment [40]. Activated inflammatory cells generate ROS which further function as chemical effectors in inflammation-driven carcinogenesis [41]. Sal-B enhanced apoptosis of cancer cells thus inhibited cancer cell proliferation and metastasis [13,42]. TNF-α is a pro-inflammatory cytokine that is involved in systemic inflammation as it can damage cell structure and increase endothelial cell permeability. Previous studies have demonstrated that Sal-B decreased LPS-induced TNF-α production in rat primary microglia in a dose dependent manner [43]. Sal-B protected endothelial cells against TNF-α-induced disruption via inhibition of VEGF and ERK activation [44]. The inhibition of NF-κB signaling could be a potential anti-inflammatory mechanism of Sal-B [43,45] as Sal-B attenuated VCAM-1 and ICAM-1 expression in TNF-alpha treated human aortic endothelial cells. Sal-B also suppressed high glucose-induced mesangial cells proliferation and extracellular matrix production through inhibition of NF-κB-dependent activation of MMP-2 and MMP-9 [46]. TNF-α has been found to have a pro-cancerous effect. Tumor cells produced TNF-α to endorse tumor cell survival through the induction of genes encoding NFκB-dependent anti-apoptotic molecules [47]. Moreover, TNF-α is implicated in tumor growth, metastasis, and angiogenesis process [5]. In 7,12-dimethylbenz(a)anthracene (DMBA)-induced squamous cell carcinoma, elevated inflammation and tumor angiogenesis in hamster were attenuated by Sal-B treatment along with decreased progression of squamous cell carcinoma [24]. Our study revealed that Sal-B reduced tumor tissue TNF-α levels induced by ESC. Thus, Sal-B may exhibit its anti-inflammatory effect via down-regulating expression of TNF-α as previously shown [8,48].

Several prognostic factors have been known to regulate cell proliferation and survival. The development of therapeutic agents that selectively target cyclin D1 is of clinical interest [49]. Our results showed that Sal-B also reduced tumor tissue cyclin D1 while it has no effect on tissue Ki-67 indicating that Sal-B might inhibit cell progression through beta-catenin pathway. Meanwhile, cisplatin has inhibitory effect on both cyclin D1 protein and Ki-67 which is consistent with previous studies reported that cisplatin down-regulated cyclin D1 [8] and Ki-67 [50]. These data suggest that Sal-B might mainly affect phase G1 phase of cell cycle and might lack significant effects on other phases of cell cycle in ESC.

Apoptosis plays a role in cancer progression and can be mediated by extrinsic and/or intrinsic pathways, where caspase expression was detected in both pathways [51]. Our findings suggest that treatment with Sal-B or cisplatin increased expression levels of tumor caspase-3 and P53 in ESC injected mice leading to cell cycle arrest. Consistent with our findings, previous study showed that Sal-B was effective for glioma through induction of apoptosis [12]. Sal-B decreased the cellular component of anti-apoptotic molecules such as NF-κB, Bcl2, and Bcl-XL and increase pro-apoptotic protein caspase-3 and P53 [52]. In triple negative breast cancer cells (TNBC), Sal-B did not change caspase-3 or caspase-8 suggesting that death-receptor (extrinsic) pathway is not involved in overall apoptotic effect whereas Sal-B inhibited Bcl-xL and survivin expression indicating the involvement of mitochondrial (intrinsic) pathway. Sal-B also inhibited ovarian cancer SKOV3 cell growth via promoting apoptosis as evidenced by increasing caspase-3 expression and blocking the cell cycle [53]. Sal-B induced cell death and triggered autophagy in HCT116 and HT29 colorectal cancer cells via suppression of AKT/mTOR pathway [54]. Sal-B also inhibited hepatocellular carcinoma cell viability in a concentration dependent manner via enhancing apoptosis and autophagy [55].

Tumor growth is also dependent on angiogenesis. Increased VEGF expression is closely associated with an increase in microvessel density [56]. Our findings suggest that either cisplatin or Sal-B lessened tumor tissue VEGF expression levels in ESC injected mice. Our data are consistent with the previous funding in oral squamous cell carcinoma [56]. Although Sal-B has shown to inhibit growth of oral squamous cell carcinoma, it had limited effects on premalignant cells [57]. The Sal-B effect was associated with inhibition of HIF-1α, TNF-α, and MMP9 suggesting that the antitumor effect of Sal-B could be attributed to its antiangiogenic effect induced by decreased expression of key regulator gene of apoptosis [57]. 

COX-2, the inducible form of COX, is undetectable in normal tissue and abundant in inflammatory and neoplastic diseases. Principle product of COX-2 is PGE2 which induces cell proliferation, promotes tumor angiogenesis, decreases immunosurveillance, and inhibits cell death. Since COX-2 is a pro-angiogenic factor in potentiating cancer metastasis [58], inhibition of COX-2, and down-stream signaling pathway could be also involved in the antiangiogenic effect of Sal-B. Overexpression of COX-2 in oral mucosa has been shown to be associated with the increased risk of head and neck squamous cell carcinoma 13. Sal-B has proven experimentally in vivo and in vitro to suppress COX-2 expression and the production of its main metabolite PGE2 in head and neck squamous cell carcinoma and these effects were associated with retarding cancer cells growth [13]. Our findings are consistent with previous findings as Sal-B significantly reduced tumor expression levels of COX-2 in ESC injected mice. Studies also showed that combined administration of Sal-B and low dose of the COX-2 inhibitor celecoxib synergistically provided profound anticancer effect via enhancing apoptosis of head and neck squamous cell carcinoma [42]. Thus, it is quite possible that COX-2/PGE2 inhibition is involved in the antiangiogenic effect of Sal-B in ESC injected mice.

## 4. Material and Methods

### 4.1. MCF-7 Cell Culture

Michigan Cancer Foundation-7 (MCF-7) human breast cancer cells were cultured in Dulbecco’s Modified Eagle’s Media (DMEM) supplemented with 10% fetal bovine serum (both from Invitrogen, Carlsbad, CA, USA) and 0.02% antibiotic (5000 units/mL of penicillin and 5000 µg/mL of streptomycin) (Thermofisher scientific, New York, NY, USA) mix at 37 °C in humidified atmosphere in a CO2 incubator.

### 4.2. Determination of Cell Proliferation Inhibition Rate by MTT Assay

5 × 103 MCF-7 cells in 200 µL of medium were inoculated in 96-well plates. Serial dilutions of sterile Sal-B ranging from 0 to 1.0 mg/mL in water were added using cells without Sal-B treatment as a positive control. After 24, 48, and 72 h culturing; 50 µL MTT at 1 mg/mL concentration was added to each well. The liquid was discarded after 4-h culturing before 150 µl of DMSO was added. The absorbance value (A) at the wave-length of 540 nm was determined by the microplate reader. Cell proliferation inhibition rate (%) = 1 − (A treated cells/A blank control) ×100%.

### 4.3. Animals Studies

Thirty female Swiss albino mice (20–25 g body weight) were used and were allowed to acclimatize to experimental conditions for three weeks. All experimental protocols and animal procedures were approved by the institutional research ethics committee at the Faculty of Pharmacy, Damanhur University, Egypt. The experiment was conducted under controlled laboratory conditions, temperature ranging from 25 ± 4 °C and a normal dark/light cycle where food and water were provided ad libitum. Thirty mice were divided into the following groups (*n* = 10/group).

- Group 1: Control Ehrlich Solid Carcinoma (ESC)—Mice Were Injected subcutaneous (S.C.) with 0.2 mL of Ehrlich Cell Line (1 × 106 Cells) Obtained from the Pharmacology and Experimental Oncology Unit of the National Cancer Institute, Cairo University, Egypt) into the Left Hind Leg.
- Group 2: Cisplatin— Mice were injected S.C. with 0.2 mL Ehrlich cell line (1 × 106 cells) into the left hind leg. On the seventh day after tumor implantation, mice received intraperitoneal (I.P.) injection of cisplatin (Sigma, St. Louis, MO, USA) as a single dose of 3.5 mg/kg, as previously prescribed [59].
- Group 3: Salvianolic acid B (Sal-B)—Mice were injected S.C. with 0.2 mL of Ehrlich cell line (1 × 106 cells) into the left hind leg. On the seventh day after tumor implantation, mice received I.P. injection of Sal-B (Sigma) 25 mg/kg [60] daily for two weeks.

### 4.4. Assessment of the Tumor Volume 

Tumor volume was measured on day 21 after ESC injection using Vernier Caliper (Mitu Toyo brand, Japan). Tumor volume was calculated using the following formula [33].

Tumor volume (mm^3^) = 4π(A/2)^2^ × (B/2)

A represents major diameter and B represents minor diameter.

### 4.5. Assessment of the Biochemical Parameters

At the end of the study, all mice were sacrificed and blood was collected to determine plasma level of malondialdehyde and reduced glutathione (GSH) spectrophotometrically according to the manufacturer instructions (Cayman, Ann Arbor, MI, USA) as markers of oxidative stress and antioxidant activity, respectively. The tumor was excised and divided into two portions: One for homogenization and the other for histopathological and immunohistochemical assessments. The isolated tumor tissue was homogenized for determination of TNF-α using a commercially available ELISA kit according to the manufacturer′s instructions (RayBiotech Inc., Norcross, GA, USA). Matrix metalloproteinase-9 (MMP-9), cyclin D1 protein, and Ki-67 protein were also assessed in homogenized tumor tissue using commercially available ELISA kits according to the manufacturer’s instructions (antibodies Inc., Atlanta, GA, USA).

### 4.6. Histopathological Examination

Tumor tissues were excised, and then fixed in 10% neutral buffered formalin. Tissues were then processed for paraffin embedding and were subsequently sectioned at 3–4 μm (Reichert Jung microtome, Wetzlar, Germany) using Augusta University histology core facility. Deparaffinized sections were stained with hematoxylin/eosin (H&E). The slides were examined for any pathophysiological changes using Zeiss M2 light microscope at 200× and 400× in a blind fashion using Dr. Abdelsayed expertise as a clinical pathologist.

### 4.7. Immuno-Histochemical Examination

Using the labelled streptavidin–biotin staining method (Zymed Laboratories Inc., San Francisco, CA) at Augusta University, immune-histochemical assessments of caspase-3, P53, VEGF (antibodies from Santa Cruz Biotechnology, CA, USA), and COX-2 (Cayman) in tumor tissue were also performed. The slides were examined by using Zeiss M2 light microscope at 200× in a blind fashion. Color intensity was quantified using Fiji software and results were presented as percentage of positive stained cells per field.

### 4.8. Statistical Analysis

All data are presented as mean ± SEM and analyzed using one-way ANOVA followed by Tukey’s post hoc test for multiple group comparisons. Following ESC injection, the percentage of mice survival was calculated in control, Sal-B treated, and cisplatin treated mice using the Kaplan–Meier method followed by the Chi-square test analysis. Analyses were performed using GraphPad Prism Version 4.0 software (GraphPad Software Inc., San Diego, CA, USA). For all comparisons, *p* < 0.05 was considered statistically significant.

## 5. Conclusions

Sal-B is a potential cytotoxic polyphenol against cancer via targeting multiple signaling pathways. Sal-B has been shown to trigger apoptosis through activation of caspases, reducing anti-apoptotic proteins (Bcl-2), activation of proapoptotic proteins (Bak, Bax), modulating PI3K/AKT/MAPK pathways, NF-κB inflammatory signaling inhibition, and ROS modulation [61]. Our data suggest that Sal-B may have a potential role to prevent the progression of breast cancer cells growth via its antioxidant, anti-angiogenic, and apoptotic effects. Although Sal-B has been shown to provide a promising chemo-preventive effect in head and neck squamous cell carcinoma, several limitations have hampered its clinical use such as poor systemic delivery and low bio-availability. Li et al. recently showed that phospholipid complex loaded nanoparticles encapsulating Sal-B provided a better cancer chemo-preventive effect against head and neck squamous cell carcinoma [62]. Although further studies are required to investigate the antitumor effect of Sal-B in other cancer cell types and determine the underlying mechanisms, it is also interesting to find out if Sal-B could be used in combination with commercially available chemotherapeutic agents to improve clinical outcome via decreasing the chance of cancer resistance and adverse effects.
